# Spontaneous Splenic Rupture due to Metastatic Pancreatic Cancer

**DOI:** 10.1155/2021/9918154

**Published:** 2021-09-04

**Authors:** D. Politis, D. Myoteri, M. Bourou, C. Nastos, I. Papaconstantinou, D. Dellaportas

**Affiliations:** ^1^2nd Department of Surgery, Aretaieion University Hospital, Medical School, National and Kapodistrian University of Athens, Greece; ^2^Pathology Department, Aretaieion Hospital, Medical School, National and Kapodistrian University of Athens, Greece; ^3^3rd Department of Surgery, National and Kapodistrian University of Athens, Attikon University Hospital, Athens, Greece

## Abstract

*Introduction*. Nontraumatic splenic rupture is a rare event. On the other hand, splenic metastasis is also rare and usually found in the context of disseminated disease. Spontaneous splenic rupture caused by a metastatic deposit has been reported only as case reports. To the best of our knowledge, pancreatic cancer being the primary site has been reported in only a handful of cases. *Case Presentation*. A case of spontaneous splenic rupture in a 68-year-old male presenting with acute onset left shoulder pain, caused by metastatic pancreatic cancer to the spleen, is presented herein. During the investigation, the patient developed hypovolemic shock due to intra-abdominal hemorrhage, necessitating emergency splenectomy. The patient recovered well and was discharged from the hospital on the 8^th^ postoperative day. *Discussion*. This study underlines the fact that the spleen is a rare site of metastasis, remaining mostly asymptomatic. However, it can nevertheless prove to be a life-threatening condition. The exact pathophysiological mechanism of splenic rupture due to metastasis still remains unknown, requiring further research. Emergency splenectomy remains the standard of care, and this clinical condition should be considered in the differential diagnosis of cases with acute abdomen and malignant neoplasm history.

## 1. Introduction

Spontaneous splenic rupture (SSR) due to metastatic deposit rupture is a rare condition. Among such cases, the primary tumor site being the pancreas is even rarer [1–3]. It is seldom considered in the differential diagnosis of the acute abdomen, especially in the absence of a previous diagnosis of cancer. A striking case of SSR managed successfully, which turned out to be caused by pancreatic metastasis, presenting as an acute abdomen is presented herein.

## 2. Case Presentation

A 68-year-old man presented to the emergency department with acute onset pain located in the back of his left shoulder. The patient had a medical history of percutaneous coronary intervention (PCI) with stent insertion in his right coronary artery and Type II diabetes mellitus. Physical examination revealed no specific findings, and his abdomen initially was soft and mildly tender in the left upper quadrant.

**Figure 1:**
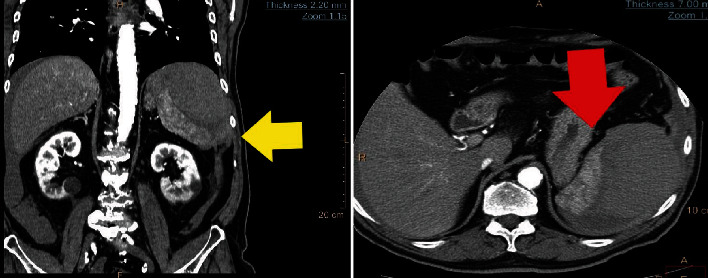
(a) Coronal CT depicting the ruptured spleen (yellow arrow) with concomitant bleeding in the left upper quadrant. (b) The ruptured spleen (red arrow) in axial CT.

Laboratory tests showed a hemoglobin level of 8.2 g/dl with the rest of the blood tests being unremarkable. While awaiting imaging investigations, the patient became restless with profound abdominal pain. He underwent computed tomography (CT) scan which revealed splenic rupture and bleeding located in the left upper quadrant (Figure 1). Shortly after the CT imaging, he became hemodynamically unstable and was emergently taken to the operating theatre.

On exploratory laparotomy, an amount of 600 ml of blood was found mostly in the left upper abdominal quadrant and the spleen was found to be ruptured. An emergency splenectomy was performed. No other macroscopic findings were noted intraoperatively.

**Figure 2:**
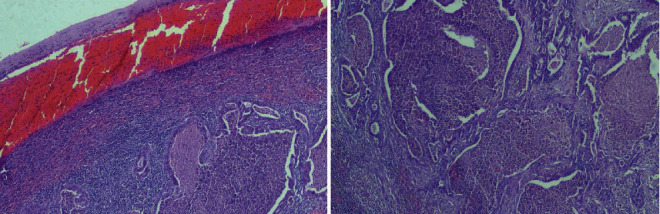
(a) Metastatic moderately differentiated adenocarcinoma just below the hemorrhage and neutrophilic infiltrate formed under the splenic capsule tear (H-E ×100). (b) Neoplastic glands with dirty necrosis in their lumina, embedded in a desmoplastic stroma with moderate inflammatory infiltration (H-E ×200).

**Figure 3:**
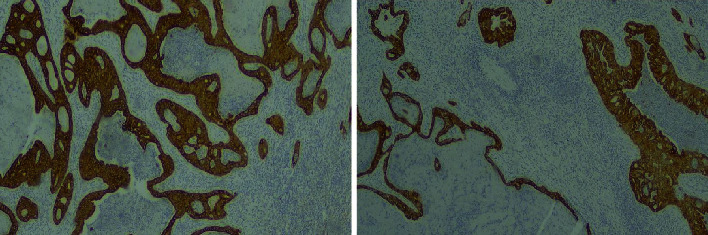
(a) Diffuse and intense cytoplasmic staining of the neoplastic glands with K7 (K7 ×200). (b) Diffuse and intense cytoplasmic staining of the neoplastic glands with K19 (K19 ×200).

Histopathological examination of the specimen showed a capsular disruption on the inferior-posterior surface of the spleen. In the bed of that capsular rupture, a tumor mass measuring 2.4 × 2.3 × 2 cm was found. Microscopically, its morphological characteristics were indicative of metastatic adenocarcinoma moderately and focally poorly differentiated, with necrosis present (Figure 2). Immunohistochemical examination results suggested that the primary site was the pancreas or biliary tract (Figure 3).

The patient recovered well and was discharged on the 8^th^ postoperative day. He underwent chest and abdominal CT scans for staging which demonstrate the primary site in the pancreatic head, with concomitant lymph nodal disease locally and in the mediastinum. The multidisciplinary team decided for palliative chemotherapy.

## 3. Discussion

The spleen is a rare site of metastasis regardless of the primary malignant neoplasm origin. Choriocarcinoma (13.3%) and melanoma (10%) are the most commonly reported primary sites with gastric carcinoma being third (6.7%) for splenic metastasis [4]. Although a small number of case reports can be found in medical literature, most data on splenic metastases is collected from cadaver studies.

The presence of splenic metastasis is mostly asymptomatic [5] with the diagnosis usually set by tomographic imaging performed for another condition or oncological staging. In cases where the splenic forming mass compresses the surroundings, symptoms such as left upper quadrant pain, shortness of breath, and early satiety may be present [6].

Metastasis to the spleen leading to splenic rupture is extremely rare. However, when patients with known splenic metastasis present with acute abdomen, it should always be considered part of the differential diagnosis [4]. Given the severity of hemorrhage due to splenic rupture, prompt diagnosis is the key to the appropriate treatment.

SSR is usually treated with emergency splenectomy regardless of the cause.

There are several theories regarding pathophysiological mechanisms of metastasis. Those theories focus on either anatomical, mechanical, or immunological factors and explain the rarity of solid tumor metastasis to the spleen. In summary, the spleen is rarely affected because of the constant blood flow to it, the rhythmical splenic sinusoid contractions, the sharp angle of the splenic artery with the celiac axis, the lack of afferent lymphatic vessels to the spleen, and the hypothesis that the splenic capsule acts as a protecting factor from intraparenchymal metastasis. Furthermore, splenic microenvironment (Kupffer cells, immunoglobulin and opsonin production, and large numbers of monocytes) and high concentration of some factors such as angiostatin make the spleen an unfavorable site for metastasis growth due to increased immune surveillance in its lymphoid cells. Considering the above, it is speculated that tumor emboli can hardly reach and implant the spleen [7, 8].

The exact mechanism of rupture of the metastasized spleen is currently unknown. Neoplastic invasion and disruption of the capsule, tumor necrosis, and subsequent bleeding as well as neoplastic infarctions leading to subcapsular rupture are the most accepted theories [4].

Considering the above, our case confirmed the fact that splenic metastases usually go unnoticed. Our patient presented with SSR, which was successfully treated with emergency splenectomy, and the fact that the spleen was metastasized was not realized until the final histopathology examination. Moreover, the primary site was suggested to be the pancreas or biliary tract, while both of them rarely metastasize to the spleen.

In conclusion, no matter how rare splenic rupture due to the presence of metastasis is, it must be considered in the differential diagnosis of an acute abdomen in patients with known cancer. More data should be collected about the pathophysiology, risk factors, and incidence of this troublesome complication.

## Data Availability

Previously reported case reports were used to support this study and are available and cited at relevant places within the text as references.
